# Antiretroviral Tenofovir Induces Senescence-Associated β-Galactosidase Activity in Primary Human Brain Vascular Cells in Multi-Layer Three-Dimensional Co-Culture

**DOI:** 10.7759/cureus.15327

**Published:** 2021-05-30

**Authors:** Rachel C Chang, Benchawanna Soontornniyomkij, Anya Umlauf, Virawudh Soontornniyomkij

**Affiliations:** 1 Psychiatry, University of California San Diego, La Jolla, USA

**Keywords:** antiviral therapy, treatment related toxicity, aging, small vessel disease, hiv, cell and tissue culture

## Abstract

Introduction

In the current context of early diagnosis of HIV infection, immediate initiation of antiretroviral (ARV) therapy, and lifelong chronic treatment, the potential ARV toxicity is of particular concern. Emtricitabine (FTC) and tenofovir (TFV) are commonly used as backbone drugs in ARV regimens recommended for initial therapy of HIV infection. Here we assessed the effects of FTC and TFV exposure on senescence-associated β-galactosidase (SA-β-Gal) activity, a marker of cellular senescence, in human brain vascular cells.

Design

Multi-layer three-dimensional cell co-cultures and *in vitro* assays.

Methods

To mimic the small vessel wall structure *in vivo*, three types of primary human brain vascular cells (endothelial cells, smooth muscle cells, and pericytes) were co-cultured on three Alvetex Scaffold disks placed on top of each other in order (three-layer three-dimensional cell co-cultures) and exposed to clinically relevant concentrations of ARV drugs (FTC, TFV, or FTC+TFV combination) or vehicle for eight days (four or five biological replicates per condition, 18 replicates totally). The SA-β-Gal activity was quantitatively assayed *in vitro* by using the chemiluminescent Galacto-Star System (T1012; Applied Biosystems, Thermo Fisher Scientific, Waltham, MA) in 54 protein lysates extracted from individual cell-culture disks. Three-factor analysis of variance (cell type, FTC, TFV) was used to assess differences in the SA-β-Gal activity levels normalized by the corresponding total protein concentrations.

Results

There was a trend for the FTC by TFV interaction effect on SA-β-Gal activity (P = 0.058). The effects of FTC and TFV were not significantly different among the three cell types. The overall effect of FTC was not significant when controlling for TFV and cell type. The overall effect of TFV was significant when controlling for FTC and cell type (F_(1,48)_ = 30.61, P < 0.001, partial η^2^ = 0.389). In the absence of FTC, TFV raised SA-β-Gal activity by 0.631 units on average, regardless of cell type (P < 0.001, partial η^2^ = 0.368). In the presence of FTC, TFV raised SA-β-Gal activity by 0.303 units on average, regardless of cell type (P = 0.015, partial η^2^ = 0.118).

Conclusion

Our preliminary findings suggest that primary human brain vascular cells exposed to TFV at clinically relevant concentrations undergo cellular senescence. This potential adverse effect of TFV should be further studied in animal models of HIV infection.

## Introduction

The widespread implementation of combination antiretroviral (ARV) therapy has decreased morbidity and mortality of HIV-infected persons, resulting in a drastic increase in their life expectancy [1]. Based on the guidelines for the use of ARV agents in adults and adolescents with HIV, the immediate initiation of ARV therapy is recommended for all HIV-infected persons, regardless of their plasma HIV RNA viral load or blood CD4+ T-cell count [2]. Since currently available ARV regimens do not cure HIV infection, ARV therapy should be continued throughout life. ARV regimens for treatment-naive HIV-infected persons generally consist of two nucleoside reverse transcriptase inhibitor (NRTI) drugs as backbones administered in combination with a third ARV drug from one of three drug classes: an integrase strand transfer inhibitor (INSTI), a non-nucleoside reverse transcriptase inhibitor (NNRTI), or a protease inhibitor (PI) with a pharmacokinetic enhancer [2].

These current ARV treatment guidelines have drawn clinical attention to the potential toxicity of long-term ARV therapy. The use of certain ARV drugs is known to be associated with dyslipidemia and other metabolic abnormalities that might increase the risk of cardiovascular disease (e.g., lopinavir/ritonavir {PI drugs} and efavirenz {an NNRTI drug}) [3,4]. In addition, efavirenz use was found to be associated with worse neurocognitive functioning in HIV-infected persons [5,6]. Clinicopathological analyses using postmortem brain samples from HIV-infected persons [7,8] suggested a potential association between the use of PI-based ARV regimens and the occurrence of cerebral small vessel disease, characterized on histopathology by vascular cell degeneration and basement membrane thickening [9,10] and commonly associated with aging, systemic arterial hypertension, and diabetes mellitus [11].

Of note, a recent clinicopathological study of HIV-infected persons by our group revealed a trend toward an association between emtricitabine (FTC, an NRTI drug) use and cerebral small vessel disease [12]. FTC is typically prescribed in fixed-dose combination with tenofovir (TFV, another NRTI drug) as backbone components of recommended initial ARV regimens for most HIV-infected persons [2]. In addition, FTC/TFV co-formulation is recommended as pre-exposure prophylaxis in individuals who do not have HIV infection but are at high risk of HIV acquisition [13].

In the current study, we aimed to assess the effects of FTC and TFV exposure at clinically relevant concentrations on cellular senescence of primary human brain vascular cells (endothelial cells {EC}, smooth muscle cells {SMC}, and pericytes {PC}). To mimic the structure of the *in vivo* small vessel wall and create an improved model of the in vivo environment, we used multi-layer three-dimensional (3D) co-cultures of EC, SMC, and PC. To assess cellular senescence, we quantitatively assayed the senescence-associated β-galactosidase (SA-β-Gal) activity in protein lysates [14-16].

The preliminary results based on two-factor ANOVA were presented as a poster at “The 2019 Student Research Showcase” and published by University of California San Diego Division of Biological Sciences in an in-house undergraduate journal “Chang RC, Soontornniyomkij B, Umlauf A, Soontornniyomkij V. The effects of antiretroviral drugs on microvascular cells: tenofovir induces cell senescence in 3D multi-layer co-culture. Saltman Quarterly 2018-2019;16:34-36.

## Materials and methods

Cell culture

Primary human brain EC (ACBRI 376) was obtained from Cell Systems (Kirkland, WA, USA). Primary human brain SMC (1100) and PC (1200) were obtained from ScienCell Research Laboratories (Carlsbad, CA, USA). These three vascular cell types were individually cultured in 5% CO_2_ incubators with complete classic medium with serum and CultureBoost (CSC, 4Z0-500, Cell Systems) in T75 flasks until Passage 7−8 for EC, Passage 5−6 for SMC, and Passage 8−9 for PC. The phenotypes of EC, SMC, and PC were verified by fluorescence immunocytochemistry for glucose transporter-1 (GLUT1/SLC2A1, rabbit monoclonal antibody, clone EPR3915, ab115730; Abcam, Cambridge, MA, USA; 1:10,000 dilution), calponin-1 (CNN1, rabbit monoclonal antibody, clone EP798Y, NB110-55650; Novus Biologicals, Centennial, CO, USA; 1:100), and platelet-derived growth factor receptor-β (PDGFRB, rabbit monoclonal antibody, clone Y92, ab32570; Abcam; 1:100), respectively. Alexa Fluor 568 goat anti-rabbit IgG (A11036; Thermo Fisher Scientific, Waltham, MA, USA; 1:500) was used as the secondary antibody. Cell nuclei were counterstained with Hoechst 33342 (H3570; Thermo Fisher Scientific; 2 μg/mL; data not shown).

Multi-layer 3D co-culture

**Figure 1:**
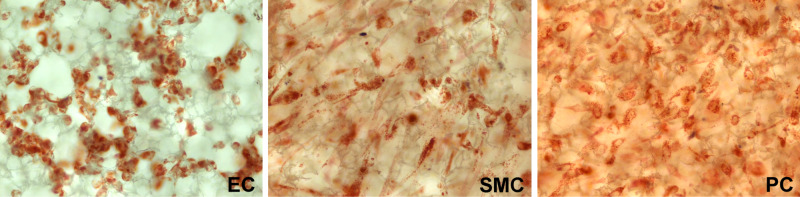
Representative images of primary human brain vascular cells in culture Endothelial cells (EC), smooth muscle cells (SMC), and pericytes (PC) cultured on Alvetex Scaffold disks are stained with Neutral Red and examined on bright-field light microscopy (original magnification x200).

For 3D cultures of vascular cells, the Alvetex polystyrene scaffold disk system (200-μm thick disk, 40-μm average void size; REPROCELL USA, Beltsville, MD, USA) was used. EC (120,000 cells in 105 μL per disk) and SMC (120,000 cells in 105 μL per disk) were individually seeded on 24-well Alvetex disks (15-mm diameter, AVP006) and PC (258,000 cells in 120 μL per disk) on six-well Alvetex disk inserts (22-mm diameter, AVP004). These three cell types were cultured with CSC for one day. The representative cell-culture Alvetex disks were stained with Neutral Red (N6264; Sigma-Aldrich, Saint Louise, MO, USA) and examined on bright-field light microscopy (Figure 1). To create multi-layer 3D co-cultures mimicking the *in vivo* small vessel wall structure, EC- and SMC-culture Alvetex disks were removed from their wells and placed on top of the PC-culture Alvetex disk inserts in the order of EC (top), SMC (middle), and PC (bottom).

To test the viability of vascular cells in multi-layer 3D co-cultures, the cells were treated with CSC with 50% medium changes made every two days. The cell-culture Alvetex disks were fixed with 4% paraformaldehyde (in phosphate-buffered saline) and stained with Hoechst 33342 for nuclear visualization. The density of well-preserved nuclei was qualitatively assessed on fluorescence microscopy (data not shown). Based on the viability of the three cell types in co-culture, the eight-day duration of ARV drug exposure was pursued.

ARV drug exposure

The multi-layer 3D co-cultures of EC, SMC, and PC were treated with CSC for one day before being exposed to ARV drugs (prepared in CSC) at the clinically relevant concentration of 2.5 μg/mL for FTC (10071; NIH AIDS Reagent Program, Division of AIDS, National Institute of Allergy and Infectious Diseases) and 0.386 μg/mL for TFV (10199; NIH AIDS Reagent Program). These concentrations of FTC and TFV were chosen based on the plasma peak concentration (C_max_, mean + 1 standard deviation) following oral administration to HIV-1 infected persons (EMTRIVA Package Insert and VIREAD Package Insert, respectively; Gilead Sciences, Foster City, CA, USA). The vascular cell co-cultures exposed to either ARV drugs (FTC alone, TFV alone, or FTC+TFV combination) or vehicle (CSC only) were maintained for eight days with 50% medium changes made every two days.

Protein extraction

The EC-, SMC-, and PC-culture Alvetex disks were removed from the inserts. To extract protein, lysis buffer supplied in the Galacto-Star System (T1012; Applied Biosystems, Thermo Fisher Scientific, Waltham, MA) was added to the individual disks and stored on ice for 10 minutes, with a 15-second vortex every three minutes. The samples were then centrifuged at 13.2K rpm for five minutes at 4°C. The supernatant protein lysates were stored at −80°C until use. The total protein concentration in each lysate sample was determined (two technical replicates) by using the Pierce BCA Protein Assay Kit (23225; Thermo Fisher Scientific, Waltham, MA).

β-Galactosidase activity assay

The SA-β-Gal activity was quantitatively assayed in protein lysates by using the chemiluminescent Galacto-Star System (T1012; Applied Biosystems) in 96-well plates in accordance with the manufacturer's protocol (two technical replicates). The chemiluminescent signal was measured at 25°C in Synergy HTX Multi-Mode Microplate Reader (BioTek, Winooski, VT, USA) and the value at 60 minutes after adding the reaction buffer (i.e., at maximal light emission) was used for data analysis. A linear standard curve was generated from serial dilutions of β-galactosidase from *Escherichia coli* (G-4635; Sigma-Aldrich). For each lysate sample, the SA-β-Gal activity level was normalized by the corresponding total protein concentration.

Statistical analysis 

In each of ARV drug exposure conditions (FTC, TFV, FTC+TFV, or vehicle), four or five biological replicates were included (18 totally). For all cell types (EC, SMC, and PC), a total of 54 lysate samples were assayed for SA-β-Gal activity. Data were analyzed using the three-factor analysis of variance (ANOVA: cell type, FTC, TFV). A two-tailed P-value of less than 0.05 was considered statistically significant. The partial eta squared (partial η^2^) measured the effect size. The statistical analyses were conducted by using IBM Statistical Package for the Social Sciences (SPSS) Statistics Version 25 (IBM, Armonk, NY).

## Results

**Figure 2:**
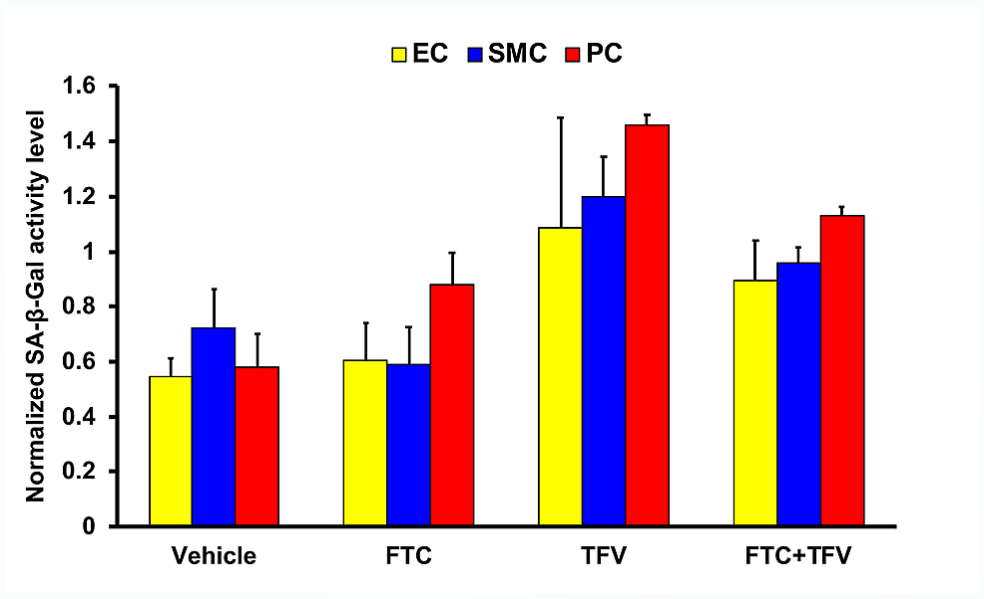
The normalized SA-β-Gal activity levels by antiretroviral drug exposure conditions The effects of antiretroviral drug (emtricitabine {FTC} alone, tenofovir {TFV} alone, or FTC+TFV combination) or vehicle exposure on senescence-associated β-galactosidase (SA-β-Gal) activity in primary human brain endothelial cells (EC), smooth muscle cells (SMC), and pericytes (PC) co-cultured on multi-layer Alvetex Scaffold disks (four or five biological replicates per experimental condition). The SA-β-Gal activity levels normalized by the corresponding total protein concentrations are shown as mean ± standard error of mean.

The SA-β-Gal activity levels normalized by the corresponding total protein concentrations in four experimental conditions (vehicle, FTC, TFV, and FTC+TFV) are shown in Figure 2. We first ran three-factor ANOVA (cell type, FTC, TFV) including all factorial interaction terms on SA-β-Gal activity. Then we reran the ANOVA by excluding the interaction terms with P > 0.10 one by one. The final run included cell type, FTC, TFV, and the FTC by TFV interaction. We found that FTC by TFV interaction effect on SA-β-Gal activity was approaching statistical significance (F_(1,48)_ = 3.79, P = 0.058). The effects of FTC and TFV were not significantly different among the three cell types (F_(2,48)_ = 2.32, P = 0.109). The overall effect of FTC was not significant when controlling for TFV and cell type (F_(1,48)_ = 1.11, P = 0.297). The overall effect of TFV was significant when controlling for FTC and cell type (F_(1,48)_ = 30.61, P < 0.001, partial η^2^ = 0.389).

Since TFV had a significant effect on SA-β-Gal activity and there was a trend for the FTC by TFV interaction effect, we chose to interpret the interaction effect from the prospective of TFV. In the absence of FTC, TFV raised SA-β-Gal activity by 0.631 units on average, regardless of cell type (P < 0.001, partial η^2^ = 0.368). In the presence of FTC, TFV raised SA-β-Gal activity by 0.303 units on average, regardless of cell type (P = 0.015, partial η^2^ = 0.118).

## Discussion

In experiments using multi-layer 3D co-cultures of primary human brain vascular cells (EC, SMC, and PC), we found that eight-day exposure to TFV at clinically relevant concentration induced an increase in SA-β-Gal activity, regardless of cell types. This finding suggests that upon TFV exposure primary human brain vascular cells undergo cellular senescence [14,15]. In contrast, the exposure to sole FTC at clinically relevant concentration showed no significant effect on SA-β-Gal activity in any of these vascular cell types. Nonetheless, there was a trend for the FTC by TFV interaction effect, that is, the effect of TFV alone on SA-β-Gal activity seemed to be stronger than that of FTC+TFV combination.

Previous *in vitro* studies examining the toxic effects of ARV drugs on vascular cells were based on traditional 2D cultures of one or two cell types [17-20]. In the current study, to mimic the *in vivo* small vessel wall more closely, we used multi-layer 3D co-cultures of EC, SMC, and PC within the Alvetex polystyrene scaffold system. This co-culture system might allow more cell-cell interaction and the vascular cells might produce their own native extracellular matrix, characteristic of the small vessel *in vivo*. Nonetheless, the current study did not attempt to verify these possibilities.

Traditionally, the SA-β-Gal activity in cultured cells is cytochemically assayed by the X-Gal staining method, which is based on the fact that lysosomal enzyme β-galactosidase catalyzes the hydrolysis of the chromogenic substrate 5-bromo-4-chloro-3-indolyl-βD-galactopyranoside (X-Gal), producing a blue precipitate that can be visualized on bright-field light microscopy [14,15]. The assessment of positive cells is conducted by counting cells stained in variable intensities. Often, it is difficult to distinguish between faint and negative staining [16]. To overcome the limitations of the X-Gal cytochemical method, in the current study we used the chemiluminescent Galacto-Star System to quantitatively assay the SA-β-Gal activity in protein lysates [16]. This chemiluminescent assay system (T1012; Applied Biosystems) was originally designed for reporter gene assays in transfected mammalian cells. Further, to account for the potential variation in protein extraction, we normalized the SA-β-Gal activity levels by the corresponding total protein concentrations.

The current study had limitations. We chose not to include HIV-1 (live particles or proteins) in the cell co-culture system because our experiments focused solely on the cytotoxic effects of ARV drugs in the ideal context of systemic viral suppression in HIV-infected persons. With regard to the assessment of cellular senescence, our findings based solely on SA-β-Gal activity assays should be further confirmed by assays of other senescence-associated markers, such as cyclin-dependent kinase inhibitor p16-INK4a [15].

## Conclusions

Based on chemiluminescent assays of SA-β-Gal activity, our preliminary findings suggest that primary human brain vascular cells in multi-layer 3D co-culture exposed to TFV at clinically relevant concentration undergo cellular senescence. To better reflect the clinical course of HIV infection and ARV therapy in humans, the chronic effects of long-term exposure to TFV can be studied in animal models such as humanized mice infected with HIV-1 and treated with ARV drugs. Further *in vitro* studies can also explore the mechanistic pathways of TFV-induced cellular senescence in human brain vascular cells. If our findings are confirmed in future *in vivo* studies, clinicians and their patients should be aware that the long-term use of TFV, one of the backbone components of recommended initial ARV regimens for most HIV-infected patients and of pre-exposure prophylaxis regimens, may lead to premature development age-related cerebrovascular disease.
